# Research on Waste Combustion in the Aspect of Mercury Emissions

**DOI:** 10.3390/ma16083213

**Published:** 2023-04-19

**Authors:** Agnieszka Kijo-Kleczkowska, Adam Gnatowski, Barbara Tora, Krzysztof Kogut, Krzysztof Bytnar, Jaroslaw Krzywanski, Dorota Makowska

**Affiliations:** 1 Department of Thermal Machinery, Faculty of Mechanical Engineering and Computer Science, Czestochowa University of Technology, 42-201 Czestochowa, Poland; a.kijo-kleczkowska@pcz.pl; 2 Department of Technology and Automation, Faculty of Mechanical Engineering and Computer Science, Czestochowa University of Technology, 42-201 Czestochowa, Poland; 3 Faculty of Civil Engineering and Resource Management, AGH University of Science and Technology, 30-059 Cracow, Poland; tora@agh.edu.pl; 4 Faculty of Energy and Fuels, AGH University of Science and Technology, 30-059 Cracow, Poland; kogut@agh.edu.pl (K.K.); bytnar@agh.edu.pl (K.B.); makowska@agh.edu.pl (D.M.); 5 Faculty of Science & Technology, Jan Dlugosz University in Czestochowa, 42-201 Czestochowa, Poland; j.krzywanski@ujd.edu.pl

**Keywords:** waste, polymer, combustion, emission, mercury

## Abstract

The topic of waste combustion/co-combustion is critical, given the increasingly restrictive legal regulations regarding its environmental aspects. In this paper, the authors present the test results of selected fuels of different compositions: hard coal, coal sludge, coke waste, sewage sludge, paper waste, biomass waste and polymer waste. The authors conducted a proximate and ultimate analysis of the materials and mercury content in them and their ashes. An interesting element of the paper was the chemical analysis of the XRF of the fuels. The authors conducted the preliminary combustion research using a new research bench. The authors provide a comparative analysis of pollutant emissions—especially mercury emission—during the combustion of the material; this is an innovative element of this paper. The authors state that coke waste and sewage sludge are distinguished by their high mercury content. The value of Hg emission during the combustion depends on the initial mercury content in the waste. The results of the combustion tests showed the adequacy of mercury release compared to the emissions of other compounds considered. Small amounts of mercury were found in waste ashes. The addition of a polymer to 10% of coal fuels leads to a reduction in mercury emissions in exhaust gases.

## 1. Introduction

The issues of waste and its cataloguing can be found in legislation [1,2]. Depending on the source of its generation, waste is divided into groups, including waste generated during the extraction of minerals, waste from agriculture, waste from paper and cardboard processing, waste from thermal processes, waste from the mechanical surface treatment of metals and plastics and municipal waste.

According to the Announcement of the Marshal of the Polish Parliament [3], waste is understood as “any substance or object which the holder discards, intends to dispose of or is obliged to dispose of”. Conversely, a waste incineration plant is intended for thermal processing, with or without the recovery of the generated thermal energy, including installations and devices used to control and monitor the aforementioned process, together with the treatment of exhaust gases released to the atmosphere.

The information on the exact amount and types of waste from plants that produce more than 1000 tonnes of waste per year and the ones that produce 1 million tonnes or more (excluding municipal waste) is presented in Ref. [4].

According to [5], regarding sludge from industrial and municipal wastewater treatment plants, sludge production per year changed as follows (in thousands of tonnes of dry matter): 1063.1 in 2000, 1124.4 in 2005, 895.1 in 2010, 951.5 in 2015, 989.5 in 2020 and 1025.8 in 2021. Meanwhile, their storage per year changed as follows (in thousands of tonnes of dry matter): 474.5 in 2000, 399.1 in 2005, 16.9 in 2010, 131.5 in 2015, 63.9 in 2020 and 84.0 in 2021. Furthermore, their thermal processing per year changed as follows (in thousands of tonnes of dry matter): 34.1 in 2000, 37.4 in 20015, 66.4 in 2010, 165.4 in 2015, 219.4 in 2020 and 221.8 in 2021. A clear reduction in sewage sludge storage with increasing thermal utilization in recent years can be observed.

The issue of waste storage requirements was examined in Ref. [6]. According to Ref. [7], one of the reference methods for testing soils on which municipal sewage sludge has been applied is the determination of the content of heavy metals—lead, cadmium, mercury, nickel, zinc, copper and chromium—by atomic absorption spectrometry (AAS) or by optical emission spectrometry with inductively coupled plasma (ICP-OES) with their prior digestion using strong acids. According to the criteria for admitting waste, coded 19 08 05 (stabilized municipal sewage sludge) [8], the acceptable limits are: loss on ignition (LOI): 8% dry weight; total organic carbon (TOC): 5% dry weight; calorific value: 6 MJ/kg dry weight.

There is an insufficient load of incineration plants in Poland, which could handle sewage sludge if only it were properly prepared for incineration, mainly in terms of a level of dewatering of >90% DS [9]. Among the available sludge drying technologies, including thermal dryers, low-temperature dryers using heat pumps and solar and hybrid dryers, solar energy has the greatest use.

One of the strategic goals presented in Ref. [10] is to eliminate the production of municipal sewage sludge as waste, which, due to its quality, poses problems with its management (in accordance with applicable regulations). This is possible due to thermal processing.

With reference to the above, the thermal utilization of sewage sludge, the need for continuous research aimed at identifying the properties and course of the waste incineration and co-incineration process have become extremely important. Given that there are 11 incineration plants operating in Poland using sewage sludge as energy fuel, further development of these technologies in Poland should be expected while taking into account the benefits of their co-combustion with other fuels. Sewage sludge, due to its properties, can be a valuable energy fuel, which has been emphasized in a number of research works, e.g., Refs. [11,12,13]. The similar calorific value of dried sewage sludge to lignite justifies the possibility of using this waste as fuel.

It should be emphasized that 86% of electricity generated in Poland is based on hard coal and lignite. The main components of coal are organic matter, mineral matter and water. The mineral substance originates from plant material from which coal seams were formed, as well as from mineral deposits mixed with plant material. Organic matter, on the other hand, mainly consists of carbon, hydrogen, oxygen, sulphur and nitrogen, as well as trace amounts of phosphorus and other compounds. Ash is a non-combustible part of the fuel, consisting mainly of a mineral substance. Together with moisture, it forms fuel ballast, reducing its quality. Thus, the calorific value of the fuel decreases with the increase in ash content. In the heating process, the organic and mineral substance of the fuel is decomposed. Moisture, numerous gases and vapours of organic substances are then released, leading to the formation of char, composed of the remains of organic matter and mineral substances of the fuel changed as a result of heating [14,15].

As the quality requirements of the coals burned in power plants increase, the issue of the combustion of highly watered fuels becomes more and more important. In order to meet the expectations of power engineers, hard coal mines were forced to expand and modernize coal enrichment plants. This causes a continuous increase in waste in the form of flotation sludge. The best way to dispose of these sludges is to incinerate them as slurries and to co-incinerate them with other materials and fuels. The issue of the thermal treatment of coal sludge has been addressed, e.g., in Refs. [16,17].

In high-temperature conditions, materials undergo thermal decomposition, which is also accompanied by a change in their physical and chemical properties. Coal fuels, in the presence of oxygen, undergo combustion and a series of stages of this process: heating, the evaporation of moisture, the degassing and combustion of volatiles and char burning [18]. These steps may be consecutive or overlapping. In the case of coal, char burnout is the stage that determines the course of the fuel combustion process. Char combustion is, in its simplest form, a high-temperature oxidation of elemental carbon to carbon dioxide [14,15].

Comparing the energy properties of biomass to coals, one can notice the same qualitative elemental composition of these fuels. However, there is a difference in quantity. The advantage of biomass is definitely the lower content of sulphur and ash compared to fossil fuels. The basic features of biomass distinguishing it from other types of fuels and having a direct impact on the course of the combustion process are the high content of volatiles, resulting in the high reactivity of the fuel, low ignition temperature, high and variable moisture content, differentiated grain size distribution of the fuel and alkali content, including potassium, sodium, chlorine and sulphur. Due to the high reactivity, the burning rate of the biomass char is definitely higher compared to that of coal. For example, the thermal decomposition of wood begins at a temperature of about 220 °C, and its individual components decompose at different temperatures, e.g., hemicellulose: 220–320 °C; cellulose: 320–370 °C; lignin: 320–500 °C. The most important gaseous components emitted from wood include CO, CO_2_, CH_4_, C_2_H_4_, C_2_H_6_ and H_2_, and the main liquid elements of the thermal decomposition of this type of fuel are water, methanol, acetic acid, acetaldehyde and tar. In general, the process of burning wood is similar to burning young coals, especially brown coals. A very large share of volatiles in this type of fuel causes over 60–70% of the calorific value of wood to be released in the degassing process. Due to the high reactivity, the burning rate of wood char is much higher compared to that of coal. The mechanism of oxidation of the wood coke residue mainly depends on the combustion temperature. At high temperatures, oxidation to CO dominates, which is then burnt in the exhaust gas. At low temperatures, however, the oxidation process to CO_2_ takes place, with the participation of oxygen sorption on the char surface [19].

The behaviour of fuels during the high-temperature process depends on their composition and the thermal and flow conditions, which also affect the emission of gases into the atmosphere [13,20,21,22,23,24,25,26]. For example, in Ref. [13], the authors emphasized that an important issue in sewage sludge incineration is the emission of pollutants such as heavy metals, mercury, dioxins, furans, NO_x_, N_2_O, SO_2_, HCl, HF and C_x_H_y_. The high nitrogen content in the sludge affects the emission of N_2_O and NO_x_. Heavy metals can be present in sewage sludge in the form of hydroxides, carbonates, phosphates, silicates and sulphates. Dioxins and furans mainly refer to compounds containing chlorinated dibenzo-p-dioxins and chlorinated dibenzofurans.

Heavy metals are monitored and controlled in combustion plants. In sewage sludge, zinc, copper, lead, chromium, nickel, manganese, cobalt, cadmium and arsenic may be present in significant amounts. Dried sewage sludge contains about 50% of ash (the main elements are Si, Fe, Al, Ca and P) and a higher proportion of volatile elements (including Na, K and P) in comparison to coal. According to the authors of Ref. [25], the behaviour of heavy metals during fuel combustion is crucial. The classification of selected metals under combustion conditions was made, with cadmium, cadmium chloride, lead chloride, lead oxide, zinc, zinc chloride, arsenic and arsenic chloride being classed as volatile; mercury and mercury chloride being classed as very volatile; cadmium oxide, chrome copper and nickel being classed as non-volatile; and lead being classed as indirect.

Table 1 shows an example share of heavy metals in fuels [26]. In relation to other materials, sewage sludge and municipal waste are characterized by a high proportion of mercury.

In Poland, there are no legal regulations regarding the permissible content of heavy metals in fuels/waste. The emission standards for waste combustion and co-combustion equipment and installations are presented in Ref. [27]. The amounts of heavy metals in exhaust gases include 0.05 mg of (cadmium + thallium)/m^3^, 0.05 mg of mercury/m^3^ and mg (antimony + arsenic + lead + chromium + cobalt + copper + manganese + nickel + vanadium)/m^3^.

According to Ref. [28], from the point of view of the management or storage of ashes, it is extremely important to analyse them in terms of the content of heavy metals. These compounds are characterized by a very long shelf life in the environment, which may result in their bioaccumulation in plants and inclusion in the biological cycle. Among all metals, the following elements should be particularly distinguished:

- cadmium destroys the kidneys, causes hypertension and influences reproductive functions, and it should also be emphasized that it poses a huge toxicological risk because it easily penetrates into ground and underground waters;
- lead and antimony destroy bones, soft tissues, liver, brain and bone marrow; and
- mercury attacks brain cells and the entire nervous system and causes paralysis [29,30].

According to Ref. [31], heavy metals can be divided with environmental protection being taken into consideration as: As, B, Cd, Hg, Mo, Pb and Se being elements of the greatest concern; Cr, Cu, Ni, V and Zn being elements of moderate concern; Ba, Co, Ge, Li, Mn, Sb and Sr being elements of less concern; Rn, Th and U radioactive elements; and Be, Sn, Te and TI being elements present in only very small concentrations.

In Ref. [32], the authors analysed the average concentrations of the heavy metals in coal, slag and fly ash samples. They found that the average concentrations of the heavy metals in the materials were:

- arsenic (As): 39.2 (17.7–56.8) mg/kg, 44.5 (37.4–50.5) mg/kg, 119.2 (73.5–217.9) mg/kg;
- lead (Pb): 10.9 (7.8–16.9) mg/kg, 25.9 (23.0–29.1) mg/kg, 40.3 (23.9–77.4) mg/kg;
- mercury (Hg): 1.3 (<0.8–1.4) mg/kg, 1.8 (1.2–2.2) mg/kg, 3.0 (1.7–3.8) mg/kg;
- chromium (Cr): 68.9 (52.3–82.2) mg/kg, 181.6 (160.0–199.1) mg/kg, 189.2 (148.4–214.3) mg/kg;
- iron (Fe): 13,445 (11,530–15,830) mg/kg, 34,839 (31,530–37,400) mg/kg, 35,211 (27,370–40,600) mg/kg;
- zirconium (Zr): 32.5 (24.4–41.8) mg/kg, 118.2 (105.3–129.7) mg/kg, 103.8 (69.8–125.2) mg/kg;
- cobalt (Co): 13.9 (<3.0–20.5) mg/kg, 25.8 (22.7–31.5) mg/kg, 22.5 (12.9–32.9) mg/kg;
- zinc (Zn): 120.2 (55.2–156.3) mg/kg, 328.2 (262.8–397.2) mg/kg, 367.6 (239.8–602.1) mg/kg;
- copper (Cu): 18.8 (13.6–23.1) mg/kg, 42.3 (39.5–45.6) mg/kg, 45.3 (32.9–52.5) mg/kg;
- nickel (Ni): 63.5 (47.1–79.0) mg/kg, 156.7 (143.2–170.0) mg/kg, 155.4 (117.3–173.3) mg/kg;
- manganese (Mn): 111.8 (73.3–141.8) mg/kg, 226.0 (207.0–254.9) mg/kg, 272.8 (214.5–312.8) mg/kg;
- vanadium (V): 145.3 (114.4–182.3) mg/kg, 278.5 (222.7–320.7) mg/kg, 374.7 (299.0–420.4) mg/kg;
- titanium (Ti): 133.1 (1132.0–1522.0) mg/kg, 3519.7 (3164.0–3982.0) mg/kg, 3568.0 (2739.0–3959.0) mg/kg.

The authors of Ref. [33] stated that the content of heavy metals in wood pellet ash produced by biomass combustion depends on the type and quality of wood biomass, the production process, the use of additives, the characteristics of the furnace, the temperature of the process, etc., and that the mean concentrations of the heavy metals they analysed are ordered as follows: Fe  >  Mn  >  Zn  >  Cu  >  Pb  >  Ni  >  Cr  >  Cd  >  Co.

In Ref. [34], the authors showed that the trend of metal concentrations was as follows: Zn > Cr > Cu > Pb > Ni > Cd for wastewater treatment plant 1; Zn > Pb > Cr > Ni > Cu > Cd for wastewater treatment plant 2; Zn > Cu > Ni > Cr > Pb > Cd for wastewater treatment plant 3; Zn > Pb > Cu > Cr > Ni > Cd for wastewater treatment plant 4; and Zn > Cu > Pb > Cr > Ni > Cd for wastewater treatment plant 5. The results were compared to the literature: according to Ref. [35], the concentrations of particular heavy metals in sewage sludge are ordered as follows: Zn > Cu > Cr > Ni > Pb > Cd.

The authors of Ref. [36] stated that the concentration of heavy metals in leaves was found to decrease in the order of Zn  >  Ni  >  Cr  >  Pb  >  Mn  >  Cu  >  Co  >  Cd, while in the roots, this was in the order of Zn  >  Ni  >  Cr  >  Pb  >  Mn  >  Cu  >  Co  >  Cd.

The concentration of selected heavy metals—chromium (Cr), manganese (Mn), iron (Fe), nickel (Ni), copper (Cu) and zinc (Zn)—in 5-year-old *Populus trichocarpa* wood was studied by Krutul et al. [37]. The average concentration of all the tested elements that can inhibit the bioethanol production process (chromium, nickel, copper and iron) was quite low in native wood and ranged between 10.0 ppm and 54.0 ppm. The average concentration of manganese and zinc in the native wood was higher, at the level of 118.0 ppm and 155.0 ppm, respectively.

The study ‘Distribution and Accumulation of Heavy Metals in Red Cedar (*Cedrela odorata*) Wood Seedling Grown in Dumpsite Soil’, by Akintola, O.O. and Bodede [38], assessed the ability of *Cedrela odorata* to accumulate and distribute heavy metals such as Cu, Pb, Zn, Cd and Co in their roots and shoots planted in dumpsite soil by determining the distribution factors and enrichment coefficients between soils and plant parts. The heavy metal concentrations before planting were 48.01–356.71 mg/kg of Cu, 28.42–26.48 mg/kg of Pb, 39.99–437.88 mg/kg of Zn, 0.69–9.59 mg/kg of Cd and 16.88–29.22 mg/kg of Co, while their concentrations after planting were 8.12–226.56 mg/kg of Cu, 11.22–227.41 mg/kg of Pb, 7.66–321.51 mg/kg of Zn, 0.31–4.78 mg/kg of Cd and 3.21–14.11 mg/kg of Co. Heavy metal concentrations (mg/kg) in roots were 9.93–20.11 mg/kg of Cu, 7.26–15.21 mg/kg of Pb, 9.05–22.35 mg/kg of Zn, 0.11–0.99 mg/kg of Cd and 4.56–6.11 mg/kg of Co and their concentration shoots of the plant were 18.01–35.22 mg/kg of Cu, 9.01–17.51 mg/kg of Pb, 18.66–37.86 mg/kg of Zn, 0.15–1.32 mg/kg of Cd and 6.45–8.01 mg/kg of Co. The study of the distribution characteristics of hazardous heavy metals in ginseng and wood-cultivated ginseng was carried out by Yang et al. [39]. Samples of ginseng and wood-cultivated ginseng were collected from 14 and 5 regions of Korea, respectively. The cultivated ginseng peels contained 40.3% of Pb, 25.9% of Cd, 47.6% of As, and 89.9% of Al. Meanwhile, heavy metals consisting of 27.2% of Pb, 28.2% of Cd, 48.3% of As and 56.8% of Al were distributed in the peels of the wood-cultivated ginseng.

The issue of mercury release from fuels and waste was addressed, for example, in Refs. [40,41,42,43,44,45,46,47,48,49,50]. In the Ref. [40], the authors analysed various types of waste: paper waste, cardboard, textiles, plastics, plastic film, sewage sludge, RDF and car tires. The mercury content in the materials was from 5 to 764 µg Hg/kg (paper waste: 10 µg Hg/kg; cardboard: 18 µg Hg/kg; textiles: 16µg Hg/kg; plastics and plastic film: 5 µg Hg/kg; car tires: 68 µg Hg/kg; RDF: 764 µg Hg/kg; sewage sludge: 564 µg Hg/kg). They revealed that mercury content in the samples results from the production process and the fractional composition. The authors characterized the behaviour of mercury in the thermal process of waste. For example, for paper waste, cardboard, RDF and sewage sludge, mercury release starts at 150 °C, and it is removed almost completely at 350 °C. The mercury in coal and biomass can be released in low-temperature processes [41,42].

According to Ref. [43], the average content of mercury in coals from Polish power plants is 112.9 µg Hg/kg, with a variety ranging from 30 to 321 µg Hg/kg. The authors stated that, along with increasing ash content in fuels, mercury content is increasing. In a mineral matter of coals, mercury is associated with pyrite and sulphates [44].

During the combustion of fuels in power plants, more than 99% of mercury is present at flame temperatures as Hg^0^; then, as the flue gas cools, some of the Hg^0^ mercury can be oxidized to Hg^2+^, combining with other flue gas components, such as SO_2_ and Cl_2_, forming the HgCl_2_ compounds Hg_2_Cl_2_ and HgS. Some of the resulting compounds can be absorbed by the fly ash that forms. Hg^0^ mercury is insoluble in water, which makes it persist in the atmosphere for up to two years. Hg^2+^ mercury is easily soluble in water, which means that it can be relatively easily removed from flue gas by means of a wet flue gas desulfurization installation [45,46].

The issue of mercury content in various fuels is also discussed in Refs. [47,48,49]. In Ref. [49], it was emphasized that during the combustion of fuels, mercury might be emitted in three main forms: as elemental mercury Hg^0^, divalent Hg^2+^ and mercury bound to ash particles.

The authors of Ref. [50] emphasized that combustion processes, in particular, those of hard and brown coal, constitute one of the main sources of ecotoxic elements emissions to the atmosphere.

The emission of ecotoxic elements into the atmosphere from combustion depends on the type of materials, their chemical composition and the process technology used [51,52].

The composition of gases generated during the combustion process is very important. Ref. [53] presents the results of experimental studies of the concentrations of the main gases after the pyrolysis process and combustion of various materials. The concentrations of gas mixture components were determined for each material at three temperatures, corresponding to the ignition, average combustion temperature and typical process temperature. In order to predict the formation of nitrogen oxides during the incineration of plastic wastes such as polyamide, it is important to quantify the composition of the gases that are generated during the primary thermal degradation [54]. In Refs. [55,56], modelling of the thermal degradation of polyamide 6 was performed. The thermal and thermooxidative degradation of polyamide 6 was investigated. The influence of oxygen in the degradation mode was demonstrated. Moreover, it explains the role of modifiers in the improvement of the fire behaviour of the blends, which reduces the flow rate of the fuel feeding the flame. Polyethylene combustion studies have been carried out in many works. In Ref. [57], the thermal degradation of pure, high-density polyethylene was studied in a cone calorimeter under ventilated conditions with a piloted ignition. The concentration and type of flue gases emitted during the combustion process were tested. Flammability and fire-resistance tests of HDPE were carried out in Refs. [58,59], and flammability and fire-resistance tests of the compounds formed during combustion were carried out in Refs. [60,61,62,63]. In Ref. [60], the thermal degradation of polyethylene was studied under a wide range of oxidative degradation conditions. The test techniques described are designed to isolate mixtures of products to minimize secondary reactions. In general, both pyrolytic and oxidative decomposition products were obtained. The pyrolysis products included a range of saturated and unsaturated hydrocarbons, which did not vary greatly in product ratio with conditions. In Ref. [61], pollutants emitted from steady-state, steady-flow gasification and combustion of polyethylene in a two-stage furnace were examined. The powdered polymer was first subjected to pyrolysis at 1000 °C and then to the combustion of its gaseous pyrolysates after mixing with air at high temperatures (900–1100 °C). The motivation for this indirect type of PE combustion was to achieve nominally pre-mixed combustion of pyrolysis gases with air, thus achieving lower pollutant emissions than those from the direct combustion of solid PE polymer. In Ref. [62], chromatographic analysis shows that, at high decomposition temperatures, pyrogas mainly consists of hydrogen, methane, ethylene and ethane, whereas at low decomposition temperatures, it mainly consists of ethylene, ethane, methane, hydrogen, propane and higher hydrocarbons. The thermal and catalytic pyrolysis of virgin high-density polyethylene (HDPE) was carried out in Ref. [63]. The result of the thermal pyrolysis showed that the product contained significant amounts of hydrocarbons. The result suggests that the oil produced by the catalytic pyrolysis of plastic waste has the potential to burn and produce combustion compounds as an alternative fuel.

Due to the harmfulness of the mercury emitted from combustion processes and the limits on such emissions in waste incineration and co-incineration facilities, the analysis of Hg emissions from the combustion of different types of waste is an important aspect of operating such installations.

## 2. Materials and Methods

The behaviour of waste during combustion depends on its composition and the conditions of the process. Coal, coal sludge, biomass waste, paper waste, coke waste, sewage sludge and polymer waste were examined during the research.

The proximate and ultimate analysis of materials, shown in Table 2, was carried out for samples with a grain size of below 200 μm, according to the Polish Standard PN-G-04502:2014-11 [64]. The tests of waste heat combustion were carried out with the use of LECO AC 500 (St. Joseph, MI, USA) apparatus, in accordance with the PN-ISO 1928:2002 [65] standards. The tests of the ash, volatile matter and moisture content in waste were carried out with the use of TGA Thermostep Eltra (Haan, Germany) apparatus, in accordance with Polish Standard PN-G-04511:1980 [66] and Polish Standard PN-G-04512:1980 [67]. The tests of the coal, hydrogen, and sulphur content in waste were carried out with the use of CHS-580 (Eltra) apparatus, using high-temperature combustion and detection in the infrared method of total sulphur content determination, in accordance with Polish Standards: PN-G-04584:2001 [68] and PN-G-04571:1998 [69].

The tests of mercury content in waste were carried out with absorptive atomic spectrometry with cold vapour (CV-AAS) generation in an automated mercury analyser: DMA-80 Milestone (Sorisole, Italy).

An interesting cognitive element was the chemical analysis of XRF, i.e., X-ray fluorescence analysis. This is a technique that allows the quantitative analysis of a material’s elemental composition. The method consists of irradiating the test sample with X-rays. The tests were carried out using Panalytical Epsilon 3XLE (Malvern Panalytical, Malvern, UK) apparatus.

For measuring the continuous combustion gas emissions during the combustion of waste, an automatic exhaust gas analyser, ECO 3000 (MRU Messgeräte für Rauchgase und Umweltschutz GmbH. Neckarsulm, Germany), was used, enabling the assessment of the emissions of carbon dioxide, carbon monoxide, sulphur dioxide, hydrogen sulphide and nitrogen oxides.

The mercury concentration in the flue gases was measured using an original bespoke measurement system. To determine the total mercury content, the following solutions were used: 10% SnCl_2_ to reduce Hg^2+^ to Hg^0^ and 10% KOH to remove acidic components. At the measurement path’s end was an EMP-3 (Nippon Instruments Corporation, Osaka, Japan) mercury detector.

**Figure 1:**
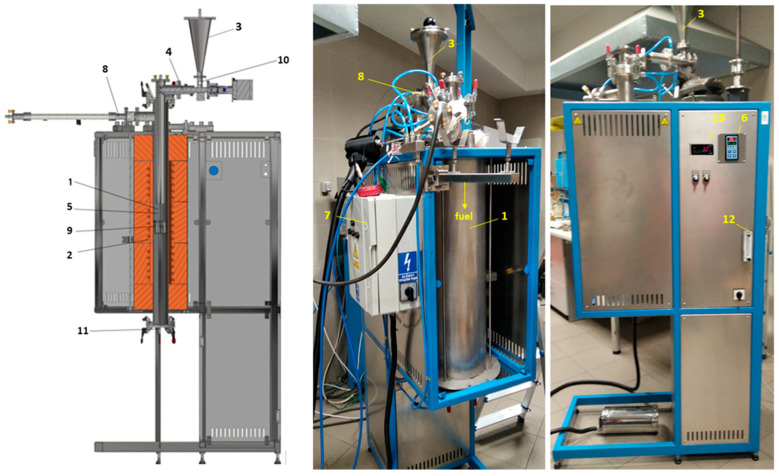
The scheme and photo of the research bench. The main element of the research bench is the combustion chamber (1), which is a pipe with a diameter of 76.1 mm, on which a three-sequence heating spiral (2) with a total power of 3.4 kW was wrapped. The pipe was thermally insulated to ensure maintenance of the desired temperature in the combustion chamber: 850 °C. Fuel/waste with a granulation of below 0.2 mm and a weight of about 30 g is served once to the charging tank (3) and then, using a screw feeder (4), to the combustion chamber. To adjust the temperature in the place of combustion (at the height of 737 mm from the feeder axis), the Ni-NiCr thermoelement was installed (5). This thermoelement is connected to the temperature regulator (6) and power controller (7). In addition, the temperature in the combustion chamber at the measurement site is controlled using the second Ni-NiCr thermoelement. The burning of waste along the height of the combustion chamber is continuous and concurrent with primary air. A connector (8) measures the gas emissions from the tested waste during combustion at the combustion chamber outlet. A container from the bottom of the combustion chamber enables the ash collection (9) in order to analyse its composition further. The sealing of detached elements is cooled using water. The total air for combustion (10 L/min) is supplied to the combustion chamber as primary air (10) and secondary air (11). The secondary air enables the burning of waste in a container of ash. The total air is measured using the rotameter (12). The spigot located behind the rotameter evenly separates the primary and secondary air. The fuel/waste is delivered to the combustion chamber using the screw feeder (4) with a rotational speed of approx. 0.63 turnover/min (13).

The tests of the materials’ combustion were carried out using a test bench (Figure 1). An interesting cognitive element was the study of the combustion of a mixture of coal fuels (90%) with polymer waste (10%).

## 3. Results and Discussion

Table 2 presents the proximate and ultimate analyses of the materials used in the research and the content of Hg in waste before combustion. It shows the diversity in their composition and heat of combustion. Polymer and biomass waste have a high content of volatile matter, while hard coal, coke waste, sewage sludge and paper waste have a high sulphur content. This is reflected in the results of the XRF analysis and the SO_3_ content in the waste samples (Table 3).

For the fuel/waste combustion tests carried out using a research bench (Figure 1), materials were used which were in the air-dry state, had a grain size of below 200 μm and had the properties presented in Table 1. Before starting the combustion measurements, the combustion chamber was heated to 850 °C, and an air stream of 10 L/min was set on the rotameter. The fuel was fed by the setting on the screw feeder controller. The average mass flow rate of each fuel/waste was about 0.067 g/s. During the combustion process, flue gas composition was measured using the ECO 3000 analyser and mercury DMA-80. The resulting ash was collected to analyse mercury content.

**Figure 2:**
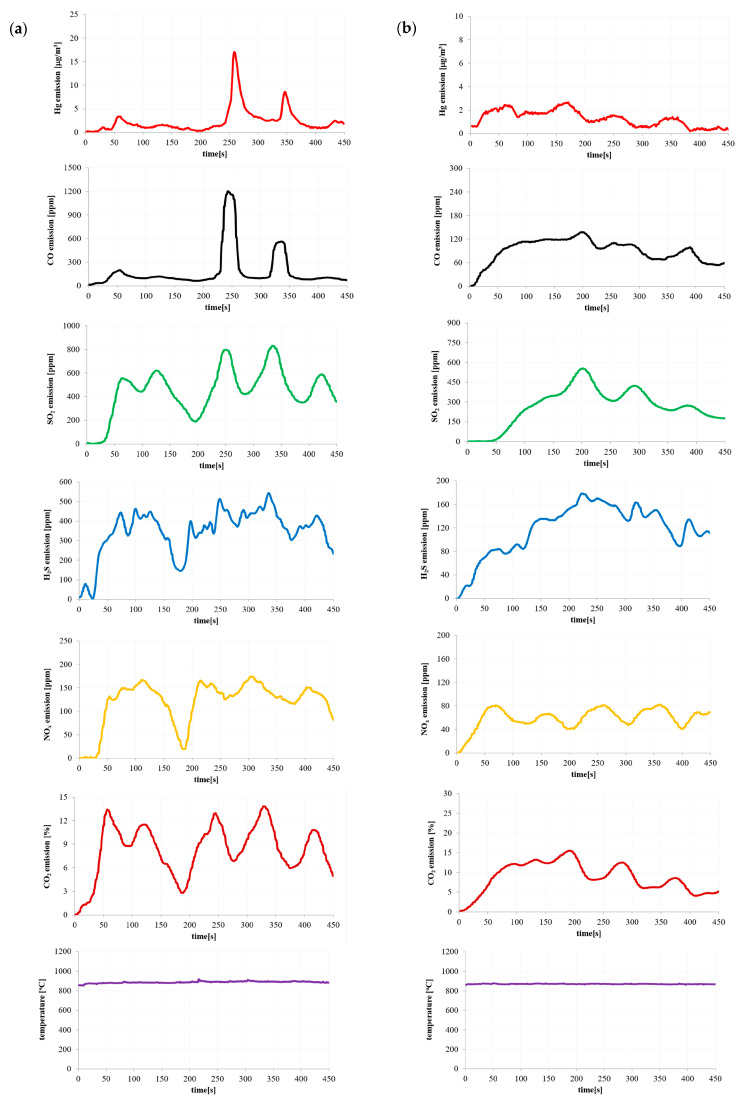
Emission of pollutants during (**a**) hard coal and (**b**) coal sludge combustion.

**Figure 3:**
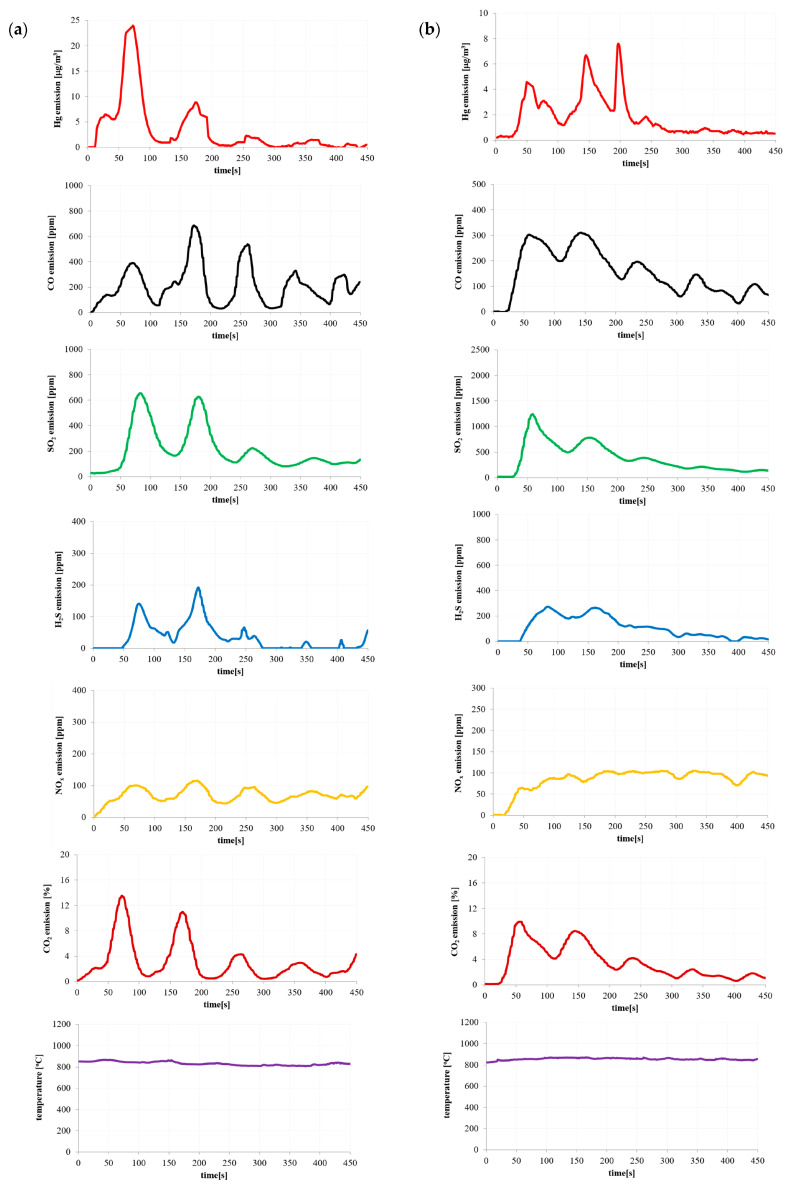
Emission of pollutants during (**a**) coke waste and (**b**) sewage sludge combustion.

**Figure 4:**
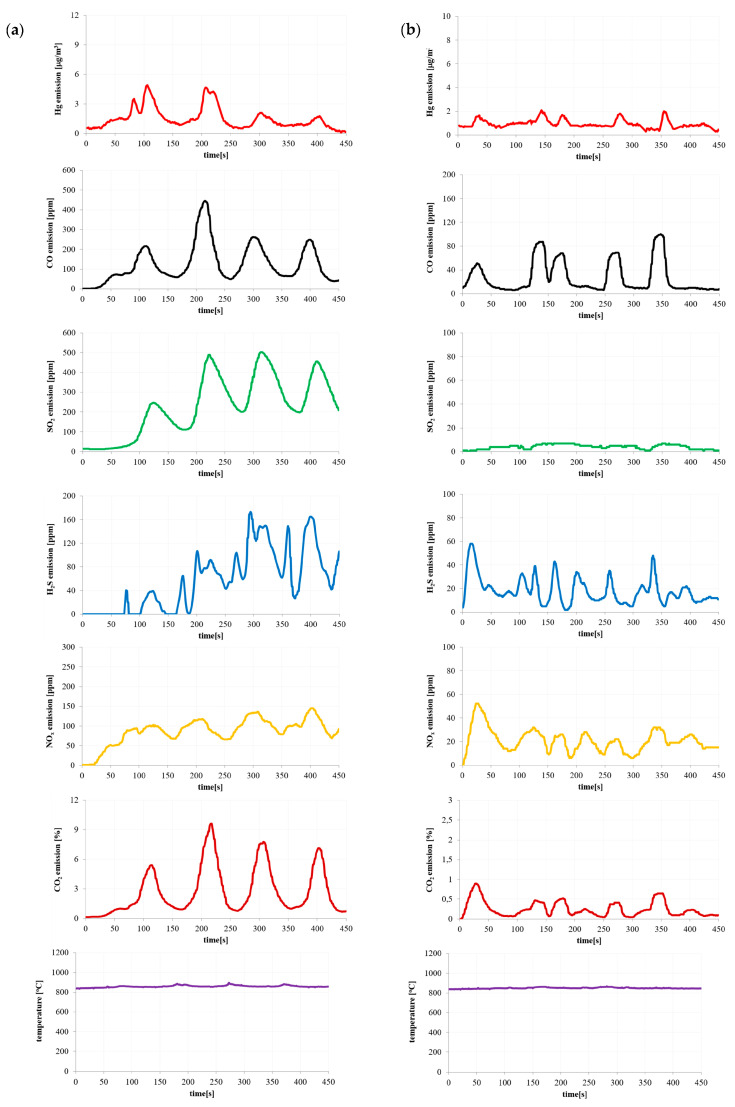
Emission of pollutants during (**a**) paper waste and (**b**) biomass waste combustion.

**Figure 5:**
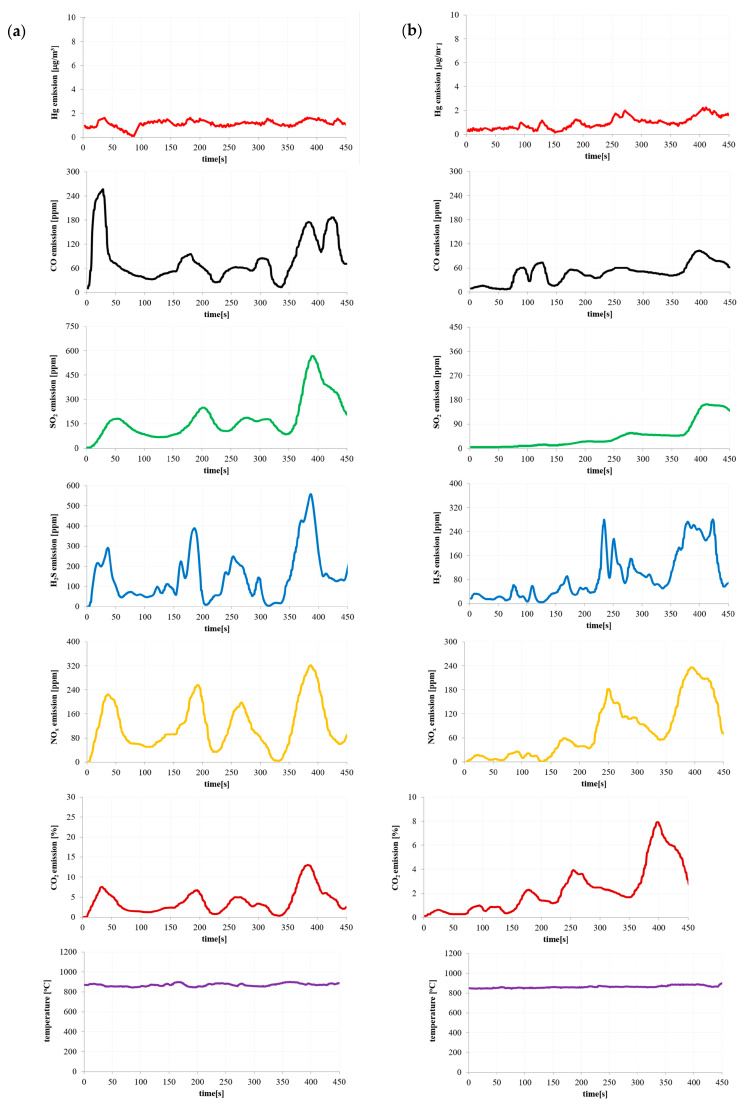
Emission of pollutants during (**a**) hard coal (90%) + polymer waste (10%) and (**b**) coal sludge (90%) + polymer waste (10%) combustion.

Based on the results of the pollutant emissions (Figure 2, Figure 3, Figure 4 and Figure 5), it can be indicated that the maximum values of mercury emission during the combustion of the considered materials are adequate in terms of their release of other considered gaseous compounds (CO, CO_2_, SO_2_, H_2_S and NO_x_). The authors also noted the adequacy of the composition of the materials in terms of the emission of gaseous compounds during combustion.

Higher NO_x_ emission values during the combustion of coal fuels–polymer mixtures result from the high nitrogen content in the considered polymer, compared to coal or coal sludge. In this paper, the authors did not determine the nitrogen content in fuels/wastes. However, the authors found nitrogen content in the considered polymer waste (N^ad^ = 15.17). It was observed that a high nitrogen content distinguishes this polymer waste from the example coal fuels and biomass (N^ad^ = 1.04 for hard coal; N^ad^ = 0.41 for coal sludge; N^ad^ = 0.53 for wheat straw [16]). This may be the reason for higher NO_x_ emissions in the combustion of a coal fuels–polymer mixture compared to the combustion of coal or coal sludge. According to Refs. [11,16], the polymer is also distinguished from coal fuels and biomass by its significant amount of hydrogen.

In this paper, during coal and coal sludge combustion, the maximum NO_x_ values were 169 ppm and 81 ppm, respectively. In the case of the combustion of a mixture of hard coal and polymer waste, this value was 319 ppm, and in the case of the combustion of a mixture of coal sludge and polymer waste this value was 235 ppm. Ref. [70] presents NO_x_ emission amounts from the combustion of various plastics in fluidized bed conditions; they found different values, even up to 233.8 ppm, which confirms the high amount of NO_x_ emissions during the incineration of polymer waste.

The temperature shown in Figure 2, Figure 3, Figure 4 and Figure 5 is the temperature in the combustion chamber during the combustion process of the presented materials. The task of the temperature controller was to constantly strive to obtain the desired temperature in the combustion chamber.

As mentioned earlier, the rotational speed of the screw feeder was kept constant throughout the measurement. However, this rotational speed was so low that it caused small masses of the tested materials to fall from the edge of the screw feeder. A similar phenomenon was observed by the authors of the paper during the measurements of a large industrial installation at very high fuel flow rates.

Table 4 presents the content of Hg in waste ash.

Depending on the tested material, we discovered that the mercury content in ashes is from 1.45 to 38.90 µg/kg Hg. We stated that coke sludge and municipal sewage sludge have the highest mercury content, both in the samples before incineration and in the ashes. In accordance with Table 5, the average emission of mercury during the combustion process relates to the content of Hg in a material’s sample before the combustion. The addition of polymer waste to coal or coal sludge decreases the average mercury emissions.

## 4. Conclusions

1. Due to the increasingly restrictive legal regulations regarding environmental aspects, it is extremely important to constantly recognize the course of coal and waste combustion, also from the point of view of the emissions of gases released into the atmosphere.
2. The mercury content of materials varies and this depends on their origin.
3. Among the materials considered, coke waste and sewage sludge are distinguished by their high mercury content: 523.16 µg/kg and 527.81 µg/kg, respectively.
4. The value of Hg emission during the combustion process depends on the initial mercury content in the waste.
5. The addition of a polymer to 10% of coal fuels leads to a reduction in mercury emissions in exhaust gases. This reduction is 53.72% in the case of hard coal (90%) + polymer waste (10%) and 26.36% in the case of coal sludge (90%) + polymer waste (10%).
6. The results of the conducted tests show the adequacy of mercury release during the considered materials’ combustion in terms of the emissions of the other compounds considered (CO, CO_2_, SO_2_, H_2_S and NO_x_).
7. The higher NO_x_ emission values during the combustion of coal fuels–polymer mixtures result from the high nitrogen content compared to coal or coal sludge in the considered polymer.
8. Under the considered process conditions, small amounts of mercury in waste ashes were found, including 6.02 µg/kg for hard coal ash, 1.45 µg/kg for paper waste ash and 6.47 µg/kg for biomass waste ash.

The tests of the considered materials’ combustion presented in the paper are preliminary studies conducted with the use of the introduced research bench. We will continue and expand our research on this aspect, including the analysis of ash.

Model research of mercury emissions can also be carried out in the future, e.g., via a fuzzy logic-based approach [71,72,73,74], which constitutes one of the leading artificial intelligence methods.

## Figures and Tables

**Table 1:** The share of heavy metals in fuels, in mg/kg [26].

Parameter	Sewage Sludge (Dry)	Municipal Waste	Beech Wood	Willow	Lignite	Hard Coal
cadmium	1.4–20	5.95	1.2	719	0.4–1.0	0.4–2.0
copper	80–800	45	1530	3.8	8–44	12–60
nickel	16–50	3	605	2.01	3–38	6–48
lead	20–50	279	185	2.91	3–24	6–50
zinc	2400–6100	663	550	135.96	3–73	20–420
mercury	2–2.5	1.22	<5	0.03	—	0.004–0.13

**Table 2:** Proximate and ultimate analyses of waste; content of mercury in materials.

Parameter/ Material	W ^ad^	V ^ad^ [%]	A ^ad^ [%]	C ^ad^ [%]	H ^ad^ [%]	S ^ad^ [%]	HHV [kJ/kg]	Hg [µg/kg]
Hard coal	12.07	29.49	6.26	51.63	10.48	1.17	21,506	216.93
Coal sludge	10.66	16.32	55.92	19.26	3.83	0.47	16,039	133.51
Coke waste	14.85	11.62	36.25	24.10	6.24	2.22	12,609	523.16
Sewage sludge	13.81	21.81	61.02	15.00	5.59	0.88	5062	527.81
Paper waste	4.33	54.80	36.69	30.19	5.20	0.64	10,740	179.78
Biomass waste	13.59	64.67	2.64	44.19	11.93	0.13	17,026	7.43
Polymer waste *	1.31	98.60	0.04	62.40	9.07	0.01	30,496	2.74

* proximate and ultimate analyses made by an external company, ^ad^—air-dried basis.

**Table 3:** The chemical analysis of XRF of materials’ samples.

Material/Chemical Compounds [%]	Hard Coal	Coal Sludge	Coke Waste	Sewage Sludge	Paper Waste	Biomass Waste	Polymer Waste
SiO_2_	25.61	44.61	17.19	28.59	20.47	18.26	17.50
Al_2_O_3_	14.31	22.77	10.46	—	11.34	10.51	10.59
Fe_2_O_3_	5.13	3.82	28.75	7.02	5.07	0.27	0.28
CaO	1.90	1.36	1.28	5.36	33.34	2.13	0.60
MgO	0.87	0.87	0.83	—	0.86	0.86	0.86
K_2_O	0.79	2.34	0.12	1.04	0.53	1.42	0.06
P_2_O_5_	0.20	0.10	4.80	15.93	2.16	0.50	0.04
SO_3_	6.88	0.87	2.96	5.88	2.53	0.10	—
MnO	0.06	0.05	0.10	0.08	0.14	0.12	0.01
SrO	0.04	0.04	0.04	0.06	0.12	0.02	0.01
TiO_2_	0.49	1.00	0.09	0.40	0.01	—	2.50

**Table 4:** Analysis of material samples during the combustion process.

Parameter/ Material	Content of Hg in Materials’ Ash [µg/kg]
Hard coal	6.02
Coal sludge	2.24
Coke waste	38.90
Sewage sludge	35.08
Paper waste	1.45
Biomass waste	6.47
Hard coal (90%) + polymer waste (10%)	4.10
Coal sludge (90%) + polymer waste (10%)	1.52

**Table 5:** The average value of emission of pollutants for the analysed materials.

Gas Emissions/Material	Hard Coal	Coal Sludge	Coke Waste	Sewage Sludge	Paper Waste	Biomass Waste	Hard Coal (90%) + Polymer Waste (10%)	Coal Sludge (90%) + Polymer Waste (10%)
Hg [μg/m^3^]	2.42	1.29	2.73	1.79	1.46	0.96	1.12	0.95
CO [ppm]	167.20	90.38	213.10	153.54	126.61	27.43	81.14	47.68
SO_2_ [ppm]	448.68	267.87	206.37	385.77	229.35	4.12	178.26	44.56
H_2_S [ppm]	348.43	118.07	33.87	108.46	57.49	18.34	148.12	90.25
NO_x_ [ppm]	119.73	59.58	69.52	84.60	87.01	20.84	120.54	78.73
CO_2_ [%]	8.31	8.77	3.05	3.51	2.71	0.25	3.83	2.28

## Data Availability

Data are contained within the article.

## References

[1] Ustawa z Dnia 14 Grudnia 2012 r. o Odpadach. https://eli.gov.pl/eli/DU/2013/21/ogl/pol.

[2] Rozporządzenie Ministra Klimatu z Dnia 2 Stycznia 2020 r. w Sprawie Katalogu Odpadów. https://eli.gov.pl/eli/DU/2020/10/ogl/pol.

[3] Obwieszczenie Marszałka Sejmu Rzeczypospolitej Polskiej z Dnia 3 Marca 2022r. w Sprawie Ogłoszenia Jednolitego Tekstu Ustawy o Odpadach. https://isap.sejm.gov.pl/isap.nsf/download.xsp/WDU20220000699/U/D20220699Lj.pdf.

[4] Rocznik Statystyczny Przemysłu. GUS, 2021. https://stat.gov.pl/obszary-tematyczne/roczniki-statystyczne/roczniki-statystyczne/rocznik-statystyczny-przemyslu-2021,5,15.html.

[5] (2022). Ochrona Środowiska 2022.

[6] Rozporządzenie Ministra Klimatu z Dnia 11 Września 2020 r. w Sprawie Szczegółowych Wymagań Dla Magazynowania Odpadów. https://eli.gov.pl/api/acts/DU/2020/1742/text/O/D20201742.pdf.

[7] Obwieszczenie Ministra Klimatu i Środowiska z Dnia 18 Listopada 2022 r. w Sprawie Ogłoszenia Jednolitego Tekstu Rozporządzenia Ministra Środowiska w Sprawie Stosowania Komunalnych Osadów Ściekowych. https://eli.gov.pl/api/acts/DU/2023/23/text.pdf.

[8] Rozporządzenie Ministra Gospodarki z Dnia 16 Lipca 2015 r. w Sprawie Dopuszczania Odpadów do Składowania na Składowiskach. https://eli.gov.pl/eli/DU/2015/1277/ogl.

[9] Suszarnie Niskotemperaturowe Czy Technologia Granulacji Osadów?. https://seidel-przywecki.eu/2020/09/03/suszarnie-niskotemperaturowe-czy-technologia-granulacji-osadow/.

[10] (2018). Strategia Postępowania z Komunalnymi Osadami Ściekowymi na lata 2019–2022.

[11] Kijo-Kleczkowska A., Środa K., Kosowska-Golachowska M., Musiał T., Wolski K. (2016). Experimental research of sewage sludge with coal and biomass co-combustion, in pellet form. Waste Manag..

[12] Kijo-Kleczkowska A., Środa K., Kosowska-Golachowska M., Musiał T., Wolski K. (2015). Mechanisms and kinetics of granulated sewage sludge combustion. Waste Manag..

[13] Werther J., Ogada T. (1999). Sewage sludge combustion. Prog. Energy Combust. Sci..

[14] Tomeczek J. (1992). Spalanie Węgla.

[15] Chomiak J. (1977). Podstawowe Problemy Spalania.

[16] Kijo-Kleczkowska A., Szumera M., Gnatowski A., Sadkowski D. (2022). Comparative Thermal Analysis of Coal Fuels, Biomass, Fly Ash and Polyamide. Energy.

[17] Nadziakiewicz J., Kozioł M. (2003). Co-combustion of sludge with coal. Appl. Energy.

[18] Kijo-Kleczkowska A., Środa K., Kosowska-Golachowska M., Musiał T., Wolski K. (2016). Combustion of pelleted sewage sludge with reference to coal and biomass. Fuel.

[19] Kordylewski W. (2005). Spalanie i Paliwa.

[20] Kosowska-Golachowska M., Luckos A., Kijo-Kleczkowska A., Musiał T., Wolski K., Środa K. (2019). Analysis of pollutant emissions during circulating fluidized bed combustion of sewage sludge. J. Phys. Conf. Ser..

[21] Li-Ming S., Shi-Suo F., Hua Z., Qi-Sheng Y., Pin-Jing H. (2013). SO_2_ and NO_x_ emissions from sludge combustion in a CO_2_/O_2_ atmosphere. Fuel.

[22] Saènger M., Werther J., Ogada T. (2001). NO_x_ and N_2_O emission characteristics from fluidised bed combustion of semi-dried municipal sewage sludge. Fuel.

[23] Shimizu T., Toyono M., Ohsawa H. (2007). Emissions of NO_x_ and N_2_O during co-combustion of dried sewage sludge with coal in a bubbling fluidized bed combustor. Fuel.

[24] Leckner B., Åmand L.-E., Lücke K., Werther J. (2004). Gaseous emissions from co-combustion of sewage sludge and coal/wood in a fluidized bed. Fuel.

[25] Hartman M., Pohořelý M., Trnka O. (2007). Behaviour of inorganic constituents of municipal sewage sludge during fluidized-bed combustion. Chem. Pap..

[26] Nadziakiewicz J. (2001). Spalanie Stałych Substancji Odpadowych.

[27] Rozporządzenie Ministra Klimatu z Dnia 24 Września 2020 r. w Sprawie Standardów Emisyjnych Dla Niektórych Rodzajów Instalacji, Źródeł Spalania Paliw Oraz Urządzeń Spalania Lub Współspalania Odpadów. https://isap.sejm.gov.pl/isap.nsf/DocDetails.xsp?id=WDU20200001860.

[28] Sushil S., Batra V.S. (2006). Analysis of fly ash heavy metal content and disposal in three thermal power plants in India. Fuel.

[29] Piecuch T. (2000). Termiczna utylizacja odpadów. Rocz. Ochr. Sr..

[30] Wilk M., Gworek B. (2009). Metale ciężkie w osadach ściekowych. Ochr. Sr. I Zasobów Nat..

[31] Vejahati F., Xu Z., Gupta R. (2010). Trace elements in coal: Associations with coal and minerals and their behavior during coal utilization—A review. Fuel.

[32] Altıkulaç A., Turhan S., Kurnaz A., Gören E., Duran C., Hançerlioğulları A., Uğur F.A. (2022). Assessment of the Enrichment of Heavy Metals in Coal and Its Combustion Residues. ACS Omega.

[33] Pazalja M., Salihović M., Sulejmanović J., Smajović A., Begić S., Špirtović-Halilović S., Sher F. (2021). Heavy metals content in ashes of wood pellets and the health risk assessment related to their presence in the environment. Sci. Rep..

[34] Kowalik R., Latosínska J., Gawdzik J. (2021). Risk Analysis of Heavy Metal Accumulation from Sewage Sludge of Selected Wastewater Treatment Plants in Poland. Water.

[35] Shrivastava S.K., Banerjee D.K. (2004). Speciation of metals in sewage sludge and sludge—Amended soils. Water. Air. Soil. Pollut..

[36] Hussain A., Priyadarshi M., Dubey S. (2019). Experimental study on accumulation of heavy metals in vegetables irrigated with treated wastewater. Appl. Water Sci..

[37] Krutul D., Szadkowski J., Antczak A., Drożdżek M., Radomski A., Karpiński S., Zawadzki J. (2021). The concentration of selected heavy metals in poplar wood biomass and liquid fraction obtained after high temperature pretreatment. Wood Res..

[38] Akintola O.O., Bodede I.A. (2019). Distribution and accumulation of heavy metals in Red Cedar (Cedrela odorata) wood seedling grown in dumpsite soil. J. Appl. Sci. Environ. Manag..

[39] Yang S.H., Lee T.W., Lee J.I., Choi H. (2019). Distribution Characteristics of Hazardous Heavy Metals in Ginseng and Wood-cultivated Ginseng. J. Food Hyg. Saf..

[40] Dziok T., Bury M., Burmistrz P. (2022). Mercury release from municipal solid waste in the thermal treatment process. Fuel.

[41] Dziok T., Kołodziejska E.K., Kołodziejska E.K. (2020). Mercury content in woody biomass and its removal in the torrefaction process. Biomass Bioenergy.

[42] Guo S., Zhang L., Niu X., Gao L., Cao Y., Wei X.-X., Li X. (2018). Mercury release characteristics during pyrolysis of eight bituminous coals. Fuel.

[43] Burmistrz P., Kogut K. (2016). Mercury in bituminous coal used in polish power plants. Arch. Min. Sci..

[44] Zhang C., Chen G., Yang T., Guoging L., Mak C., Kelly D., Xu Z. (2007). An Investigation on Mercury Association in an Alberta Sub-bituminous Coal. Energy Fuels.

[45] Senior C., Helble J., Sarofim A. (2000). Emissions of mercury, trace elements, and fine particles from stationary combustion sources. Fuel Process. Technol..

[46] Wichliński M. (2017). Emisja rtęci z polskich elektrowni w świetle konkluzji BAT. Polityka Energetyczna.

[47] Kogut K., Gorecki J., Burmistrz P. (2021). Opportunities for reducing mercury emissions in the cement industry. J. Clean. Prod..

[48] Dziok T., Bury M., Bytnar K., Burmistrz P. (2021). Possibility of using alternative fuels in Polish power plants in the context of mercury emissions. Waste Manag..

[49] Wichliński M., Kobyłecki R., Bis Z. (2011). Emisja rtęci podczas termicznej obróbki paliw. Polityka Energetyczna.

[50] Makowska D., Wierońska F., Dziok T., Strugała A. (2017). Emisja pierwiastków ekotoksycznych z procesów spalania paliw stałych w świetle regulacji prawnych. Polityka Energetyczna Energy Policy J..

[51] Lorenz U. (2005). Skutki Spalania Węgla Kamiennego Dla Środowiska Przyrodniczego i Możliwości Ich Ograniczania.

[52] Burmistrz P., Kogut K., Marczak M., Zwaździak J. (2016). Lignites and subbituminous coals combustion in Polish power plants as a source of anthropogenic mercury emission. Fuel Process. Technol..

[53] Kropotova S.S., Kuznetsov G.V., Strizhak P.A. (2022). Identifying products of pyrolysis and combustion of materials at incipient stages of fires. Fire Saf. J..

[54] Leichtnam J.N., Schwartz D., Gadiou R. (2000). The behaviour of fuel-nitrogen during fast pyrolysis of polyamide at high temperature. J. Anal. Appl. Pyrolysis.

[55] Siat C., Bourbigot S., Bras M. (1997). Thermal behaviour of polyamide-6-based intumescent formulations—A kinetic study. Polym. Degrad. Stab..

[56] Dabrowski F., Bourbigot S., Delobel R., Bras M. (2000). Kinetic modelling of the thermal degradation: Of polyamide-6 nanocomposite. Eur. Polym. J..

[57] Luche J., Mathis E., Rogaume T., Richard F., Guillaume E. (2012). High-density polyethylene thermal degradation and gaseous compound evolution in a cone calorimeter. Fire Saf. J..

[58] Zhang S., Chen H., Zhang Y., Zhang Y.-M., Kan W., Pan M. (2020). Flame Retardancy of High-Density Polyethylene Composites with P,N-Doped Cellulose Fibrils. Polymers.

[59] Shi L., Gong J., Huang D., Liu X., Zhang G. (2018). Numerical Study on the Spontaneous Combustion of High-density Polyethylene. Procedia Eng..

[60] Hodgkin J.H., Galbraith M.N., Chong Y.K. (2006). Combustion Products from Burning Polyethylene. J. Macromol. Sci. Part A Chem..

[61] Gonçalves C.K., Tenório J., Levendis Y.A., Carlson J.B. (2008). Emissions from the Premixed Combustion of Gasified Polyethylene. Energy Fuels.

[62] Frolov S.M., Shamshin I.O., Kazachenko M.V., Aksenov V.S., Bilera I.V., Ivanov V.S., Zvegintsev V.I. (2021). Polyethylene Pyrolysis Products: Their Detonability in Air and Applicability to Solid-Fuel Detonation Ramjets. Energies.

[63] Anene A.F., Fredriksen S.B., Sætre K.A., Tokheim L.-A. (2018). Experimental Study of Thermal and Catalytic Pyrolysis of Plastic Waste Components. Sustainability.

[64] (2014). Węgiel Kamienny i Brunatny—Pobieranie i Przygotowanie Próbek do Badań Laboratoryjnych—Metody Podstawowe.

[65] (2002). Paliwa Stałe—Oznaczanie Ciepła Spalania Metodą Spalania w Bombie Kalorymetrycznej i Obliczanie Wartości Opałowej.

[66] (1980). Paliwa Stałe—Oznaczanie Zawartości Wilgoci.

[67] (1980). Paliwa Stałe—Oznaczanie Zawartości Popiołu Metodą Wagową.

[68] (2001). Paliwa Stałe—Oznaczanie Zawartości Siarki Całkowitej i Popiołowej Automatycznymi Analizatorami.

[69] (1998). Paliwa Stałe—Oznaczanie Zawartości Węgla, Wodoru i Azotu Automatycznymi Analizatorami—Metoda Makro.

[70] Jankowski D., Żukowski W. (2011). Procesy termiczne jako metody redukcji odpadów z tworzyw sztucznych—Badania wstępne w reaktorze ze złożem fluidalnym. CHEMIK.

[71] Krzywanski J., Czakiert T., Zylka A., Nowak W., Sosnowski M., Grabowska K., Skrobek D., Sztekler K., Kulakowska A., Ashraf W.M. (2022). Modelling of SO_2_ and NO_x_ Emissions from Coal and Biomass Combustion in Air-Firing, Oxyfuel, iG-CLC, and CLOU Conditions by Fuzzy Logic Approach. Energies.

[72] Krzywanski J. (2019). Heat Transfer Performance in a Superheater of an Industrial CFBC Using Fuzzy Logic-Based Methods. Entropy.

[73] Krzywanski J., Urbaniak D., Otwinowski H., Wylecial T., Sosnowski M. (2020). Fluidized Bed Jet Milling Process Optimized for Mass and Particle Size with a Fuzzy Logic Approach. Materials.

[74] Krzywanski J., Czakiert T., Nowak W., Shimizu T., Zylka A., Idziak K., Sosnowski M., Grabowska K. (2022). Gaseous emissions from advanced CLC and oxyfuel fluidized bed combustion of coal and biomass in a complex geometry facility: A comprehensive model. Energy.

